# Influence of precipitation and temperature on maize production in the Czech Republic from 2002 to 2019

**DOI:** 10.1038/s41598-021-89962-2

**Published:** 2021-05-17

**Authors:** Mansoor Maitah, Karel Malec, Kamil Maitah

**Affiliations:** Faculty of Economics and Management, Czech University of Life Sciences Prague, Kamýcká 129, Prague, 16500 Czech Republic

**Keywords:** Climate sciences, Environmental social sciences

## Abstract

Maize is one of the important food crops in the Czech Republic, its growth and productivity are influenced by climate change. This study investigated the influence of precipitation under recent climate change on maize yield both for grain and silage in the whole Czech Republic during 2002–2019. Total maize yield and yield rate increased in the Czech Republic from 1961 to 2010, but they became to decrease after 2010. This is in line with the tendency of decreased precipitation and an increase in temperature after 2010, and changes are especially significant during the maize growing period, which indicates the importance of temperature and precipitation. In detail, there is a low to moderate negative correlation (−0.39 to −0.51) between grain maize yield and the average temperature in August for almost all the regions. While there is a low negative correlation between silage maize yield with the average temperature in July and August from some regions. The precipitation in July exhibited moderate to high positive correlation (0.54–0.79) to grain maize yield rate for almost all the regions, and it had low to moderate positive correlation (0.35–0.70) to silage maize yield rate for all the regions. Water deficit exhibited a negative correlation with both maize yield rate and its influence mainly in July for silage but both in July and August for grain. Farmer’s profit from grain maize is influence by yield rate, temperature, precipitation, and water deficit. A positive correlation was found between profit and grain yield rate and precipitation from July and August, while a negative correlation was detected between profit and water deficit and the average temperature in July and August. In conclusion, our results pointed out the factors influencing maize yield rate under changing climate conditions in the Czech Republic, and it warrants further studies on how to maintain maize production in a changing climate.

## Introduction

Cereals (maize, rice and wheat) are of vital importance for humans, and their world annual demand is expected to over 3.3 billion tonnes by 2050, which is 600 million tonnes more than 2019^1^. Maize is one of the world’s most widely produced and consumed cereal crops, and its global production reached 1.1 billion tonnes in 2019^2^. Besides its primary use for food, it’s also used in the industry to produce biofuels, to extract starch, oil and other substances for industrial uses. However, the rising demand for maize and declining productivity could triple the developing world’s maize imports by 2050. Therefore, the Food and Agriculture Organization (FAO) proposed the ‘Save and Grow’ model of crop production intensification aims at increasing both yields and nutritional quality while reducing costs to farmers and the environment^1^.

Europe is one of the world's biggest and most beneficial providers of food and fiber. In 2008, it represented 20% of worldwide cereal production. About 63% of the European cereals are produced in the EU27 nations. The profitability of European farming is by and large high, specifically in Central Europe, and normal cereal yields in the EU nations are over 60% higher than the world normal^3^. Intensive cultivating frameworks in central Europe, by and large, have a low sensitivity to environmental and climate change, on the grounds that a given change in temperature or precipitation has an unobtrusive effect^4^. Concentrated frameworks in cool environments may along these lines react well to unobtrusive climatic warming^3^. Then again, a portion of the cultivating frameworks as of now situated in sweltering and dry territories are required to be most seriously influenced by environmental and climate change^5^. There is a huge variety across the European mainland in climatic conditions, soils, land use, framework, political and monetary conditions^6^.

Maize is one of the most important crops in the Czech Republic, and it is grown both for silage and food. The Czech Republic is a net importer for maize with annual growth of world imports of 5%, and an annual increase in world market share is −13.33%. The value of maize (excluding seed for sowing) imported in 2019 is 42,800 thousand USD while a trade balance in 2019 is −14,601 thousand USD^7^. In the Czech Republic, the interest in cultivating maize increased dramatically since 1990 (land for maize production increase from 44,941 ha in 1990, to 75,853 ha in 2019) with its total yield (t) has grown over fivefold and the unit yield (t/ha) increased about threefold. This is driven by one of the Czech Ministry of Agriculture’s aims to ensure food self-sufficient by 2030 concerning climate change.

Climate change is a global phenomenon, but its impact is different based on each region. In Europe, less precipitation in summer and rising temperatures will lead to more frequent and intense summer droughts. The Mediterranean region is already experiencing these effects and is expected to suffer from more extreme droughts in the coming decades, together with other regions, such as central Europe. Water availability and drought have become one of the main factors affecting field crop yields and variability in the Czech Republic^8^. The effect of climate change will be evident in the field of agriculture especially when it comes to crop yields and the location where these crops can be grown. For example, the climatic variability influences crop (winter wheat and spring barley) yield variability in the Czech Republic is more now than it did in the late 19th and early twentieth centuries^9^. The extreme weather events such as especially heat waves, droughts, and heavy precipitation are already shown to cause crop failure and influence crop yield variability in the Czech Republic as well as other European countries over the centuries^10–12^.

The average global temperatures have increased, while the U.S corn belt has experienced a decrease in temperatures; thus, leading to a boost of corn yield^13^. On the other side of the Atlantic, France and Romania holding the front for corn production in Europe with 21% and 13%, respectively. European Union has presented a steady growth after the drastic decrease in 2015 of 25%^14^. One feature remains true for all regions meaning that maize production declines when extreme temperatures occur during pollination and the same happens during periods of water deficits. Tigchelaar et al. (2018) combined empirical models of maize production with future warming scenarios, and it shows that maize yields are foreseen to decline by 20–40% and 40–60% with a temperature increase of 2 and 4 °C, respectively^15^. Meanwhile, a statistical model was used to estimate the effects of heat stress and drought on annual maize production, variability, and trend from the country level to the global scale, and heat stress and drought can explain 50% of the observed global production variability in the period 1980–2010. Furthermore, the model shows global maize losses, due to extreme climate events with a 10‐year return (1980–2010), which will become the new normal already at 1.5 °C global warming levels (approximately 2020s). At 2 °C warming (the late 2030s), maize areas will be affected by heat stress and drought never experienced before, affecting many major and minor production regions^16^.

In this decade (2011–2019), the average annual temperature increased by 0.7 °C and average annual precipitation decreased by 88 mm compared to the last decade (2001–2010) in the Czech Republic. Meanwhile, the maize yield rate slightly decreased from 7.73 t/ha (2001–2010) to 7.67 t/ha (2011–2019). This decrease can be significant when considering the improvement of technological and management on maize production. Therefore, the objective of this study is to (1) explore the influence of precipitation on current maize production (for grain and silage) in the Czech Republic; (2) investigate the influence of climate change (especially temperature, precipitation, and water deficit) on maize yield rate in the Czech Republic; (3) evaluate farmer’s income from maize harvest under climate change (especially temperature, precipitation, and water deficit)impact.

## Material and methods

### Study area

**Figure 1:**
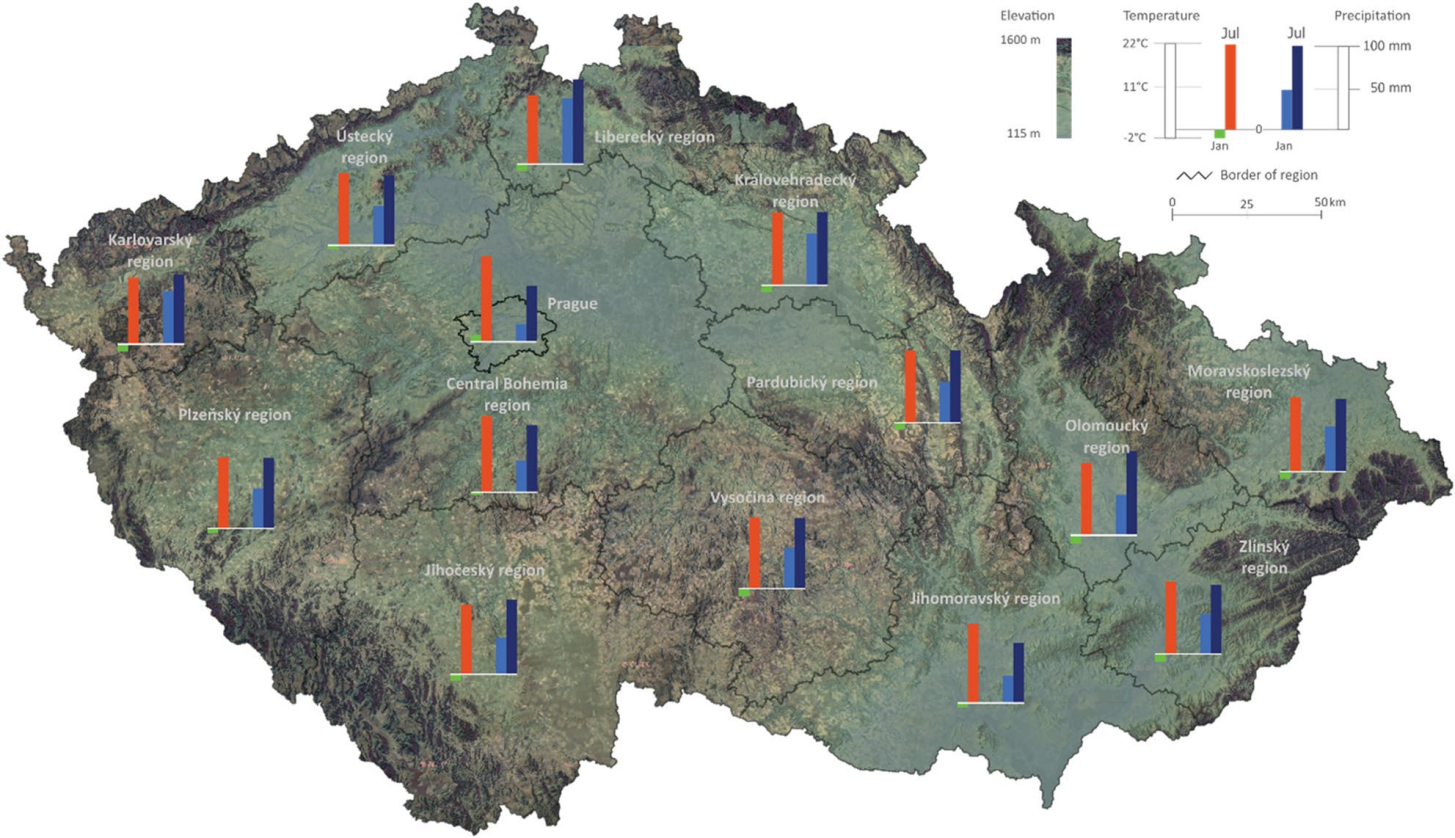
Map of the Czech Republic with annual temperature and precipitation in January and July.

The Czech Republic belongs to central Europe, and it lies mostly between latitudes 48° and 51°N and longitudes 12° and 19°E. It has typical European continental influenced temperate climate with warm, dry summer and fairly cold winter. Temperatures vary depending on the elevation with temperatures decrease while precipitation increases at higher altitudes. The highest temperature normally in July, followed by August and June, and most precipitation during the summer period. More detailed information is shown in Fig. 1.

### Data collection and analysis

The data of maize yields and cost for maize production (both for grain and silage) in the Czech Republic including 14 regions from 2002 to 2019 were obtained from the Institute of Agricultural Economics and Information (IAEI, http://www.iaei.cz). The temperatures and rainfall data from 1961 to 2019 come from the Czech Hydrometeorological Institute (CHMI, https://www.chmi.cz/historicka-data/pocasi/zakladni-informace?l=en). The climate maps for temperature and precipitation are obtained from the CHMI as well. The grain maize yield from 1920–2019 was collected from the Czech Statistical Office (https://www.czso.cz).

### Water deficit calculation

Water deficit is a very important parameter for crop production. We made the water deficit calculation for maize (both for grain and silage, 2002–2019) based on the Czech technical norm (ČSN 750,434, http://www.technicke-normy-csn.cz/750434-csn-75-0434_4_15406.html), which uses standardized temperatures (ST) according to the long term averages. Standardized temperatures are devoted to optimal rainfalls (OR) which are stated for the vegetation period (April—October) and they represent the sum of monthly rainfalls that ensure maximal yield for a given crop. In this study, we calculated the water deficit for grain from May to September, and silage from May to August. The obtained results can be negative values when there was a recorded water surplus, or positive if there was a lack of rainfall. The water deficit (DW) for maize is calculated according to the following equations.

1 $$\mathrm{DW}=\mathrm{WB}*\mathrm{A}$$

where DW = water deficit, WB = water balance, A = harvest area of maize

2 $$\mathrm{WB}=\mathrm{r}-\mathrm{AOR}$$

where WB = water balance, r = observed rainfalls for given period, AOR = Adjusted optimal rainfalls.

3 $$\mathrm{AOR}=\mathrm{aor}+\mathrm{ OR}$$

where AOR = Adjusted optimal rainfalls, aor = adjustment for optimal rainfalls, OR = optimal rainfalls

4 $$\mathrm{aor}=\mathrm{td}*5$$

where aor = adjustment for optimal rainfalls, td = temperature difference between optimal and observed values.

5 $$\mathrm{td}=\mathrm{ROT}-\mathrm{TS}$$

where td = temperature difference between optimal and observed values, ROT = Rounded observed temperatures (to the whole number), TS = Temperature standard (long term average).

### Data analysis

The mean temperature and precipitation were calculated for each decade from 1961 to 2019. The average maize yield rate was calculated for 2002–2010 and 2011–2019. A T-test was run to compare the difference of maize yield rate between 2002–2010 and 2011–2011 for the 14 regions of the whole Czech Republic. Pearson correlation was carried out to explain the effects of temperature, precipitation, and water deficit on maize production and profit. The profit (CZK/ha) was calculated by the difference between total income (yield rate (t/ha) X maize price (CZK/t)) and cost (CZK/t), without taking subsidy into account. The correlation coefficient r absolute value between 0.7 and 0.9 is considered as high correlation, 0.5–0.7 as moderate correlation, 0.3–0.5 as low correlation, and 0–0.3 as negligible correlation. All the statistical analyses and plots were realized by Origin 2019b. The significant difference was set as p < 0.05.

## Results and discussion

### Maize yield trends

**Figure 2:**
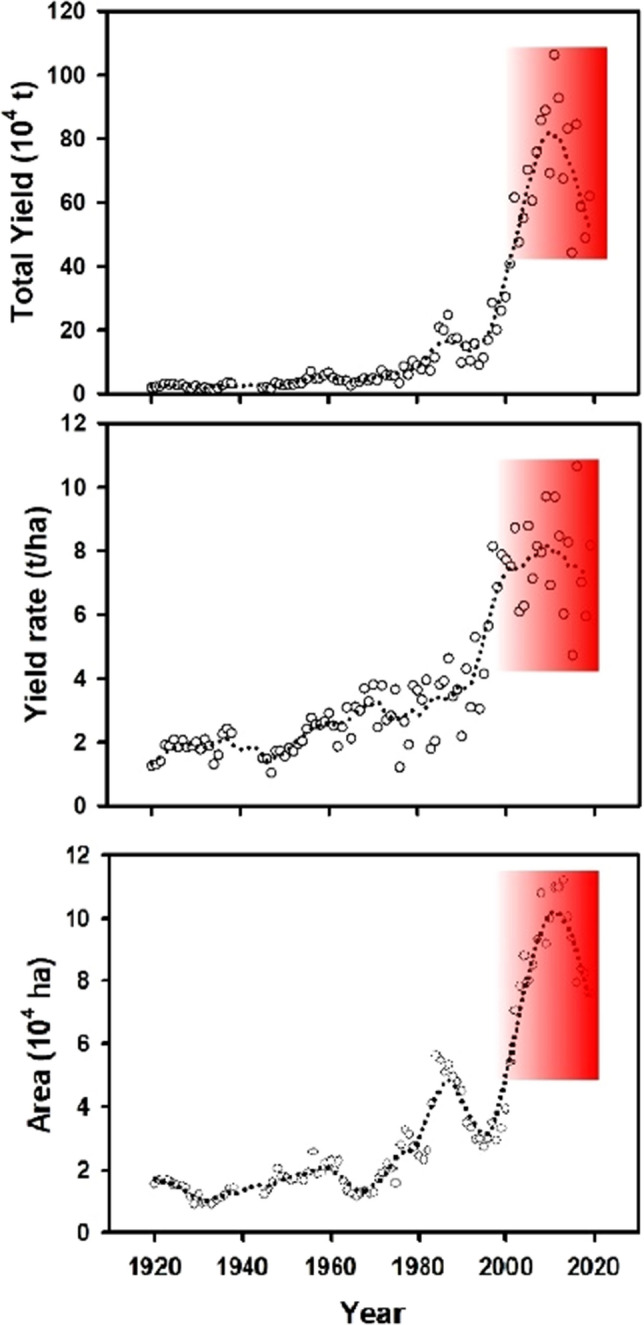
Grain maize total yield and yield rate in the Czech Republic during 1920–2019. The dotted curve is polynomial regression and weights computed from the Gaussian density function. The red area highlights the change in year 2000–2019.

In the Czech Republic, both grain maize yield and yield rate slightly increased from 1920 to 1960, and a faster increase since the 1960s, and a dramatic increase in the late 1990s (Fig. 2). The mean grain maize yield rate is 2.9 t/ha and 7.73 t/ha in the 1960s (1961–1970) and 2010s (2001–2010), respectively. This means an average annual increase rate of 96.65 kg/ha over 50 years (1961–2010), which is comparable with the situation in the USA (100 kg/ha over the last 60 years of the last century from 1 to 7 t/ha). This is mostly driven by the improvement of cultivation technology and management levels^17^. However, both grain maize yield and yield rate exhibited a sharp decrease since 2010. The grain maize yield rate slightly decreased from 7.73 t/ha (2001–2010) to 7.67 t/ha (2011–2019). This decrease can be significant when considering the improvement of technological and management on maize production, and it will have a significant influence on food self-sufficiency. For instance, even a 5% yield decline of silage maize would lead to its shortage of animal feeding in the Czech Republic^18^. Therefore, a more detailed analysis of the change was evaluated for each region during 2002–2019.

Since the 1990s, the interest in grows silage maize increased in the Czech Republic. Thus, we include both grain and silage maize for detailed changes from 2002 to 2019. The South Moravian region is the most productive region for grain maize which accounts for 42% (average production of 38,525 t) of the whole Czech Republic, and Central Bohemia region is the second with about 14% average production of 12,857 t) of the whole country. The top three regions for silage maize production are Vysocina, South Boheimia, and Central Boheimia with an average yield of 33,371 t (15.8%), 31044t (14.7%), and 30652t (14.5%), respectively. The change of yield rate is significant on silage maize in which all the regions achieved significantly (all p < 0.05) higher yield rate in 2011–2019 than in 2002–2010 (Table 1). On the contrary, there is an insignificant (all p > 0.05) difference in grain maize yield rate between 2011–2019 and 2002–2010 (Table 1).

**Table 1:** Comparison of maize (grain and silage) mean yield rate (t/ha) between 2002–2010 and 2011–2019 in the Czech Republic, the standard deviation in the bracket.

	Grain maize yield (t/ha)			Silage maize yield (t/ha)		
	2002–2010	2011–2019	p	2002–2010	2011–2019	p
Prague	7.4 (1.1)	8.4 (1.6)	0.175	31.3 (3.2)	37.9 (4.2)	**0.002**
Central Bohemia	7.1 (1.1)	7.3 (2.1)	0.816	32.1 (3.4)	38.1 (4.2)	**0.005**
South Bohemia	4.4 (1.5)	3.5 (1.9)	0.293	33.8 (3.9)	38.4 (4.8)	**0.043**
Pilsen	5.9 (1.4)	5.1 (2.5)	0.417	33.3 (3.8)	38.3 (4.7)	**0.029**
Karlovy Vary	3.6 (1.6)	2.8 (1.5)	0.271	33.9 (3.8)	38.4 (4.8)	**0.046**
Ústí nad Labem	6.8 (1.0)	7.1 (1.8)	0.604	32.0 (3.2)	38.1 (4.1)	**0.004**
Liberec	4.9 (1.4)	4.2 (1.9)	0.411	33.4 (3.7)	38.3 (4.6)	**0.027**
Kralové hradecký	5.7 (1.0)	5.9 (1.4)	0.735	32.4 (3.1)	38.1 (4.1)	**0.005**
Pardubice	5.8 (1.1)	5.6 (1.9)	0.794	32.8 (3.4)	38.2 (4.3)	**0.011**
Vysočina	4.2 (1.5)	3.4 (1.8)	0.299	33.8 (3.8)	38.4 (4.7)	**0.041**
South Moravian	7.1 (1.0)	7.7 (1.7)	0.357	31.7 (3.2)	38.0 (4.2)	**0.003**
Zlín	5.2 (1.0)	5.3 (1.3)	0.782	32.6 (3.1)	38.1 (4.1)	**0.006**
Moravian-Silesian	4.8 (1.2)	4.5 (1.5)	0.642	33.1 (3.4)	38.3 (4.3)	**0.015**
Olomouc	5.8 (1.0)	6.2 (1.4)	0.588	32.3 (3.1)	38.1 (4.1)	**0.004**

### Changes in precipitation and temperature

**Figure 3:**
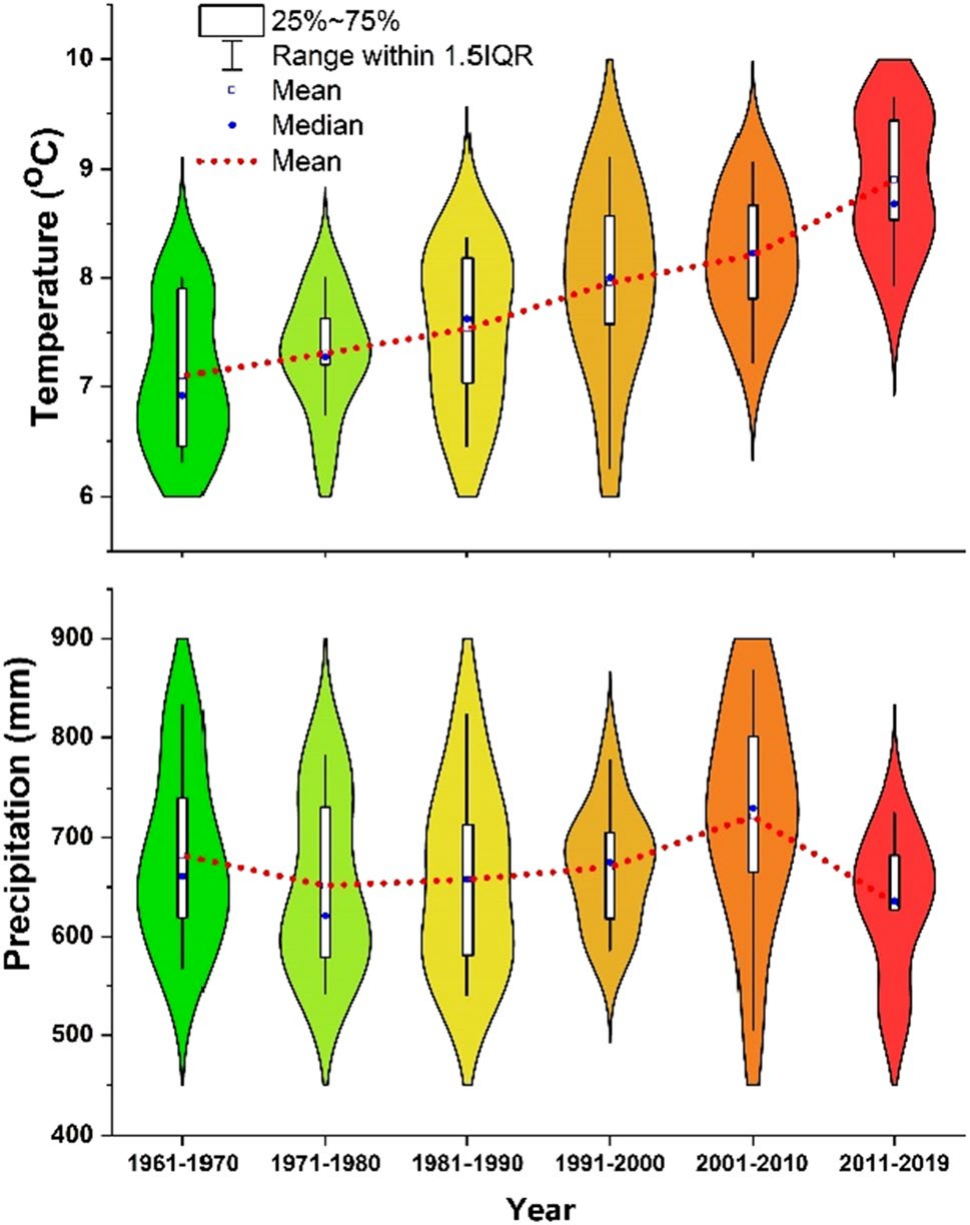
Precipitation and temperature at each decade in the Czech Republic during 1961–2019. The violin plot with boxplot shows both the distribution of the data and its summary statistics (mean/median and interquartile ranges).

**Figure 4:**
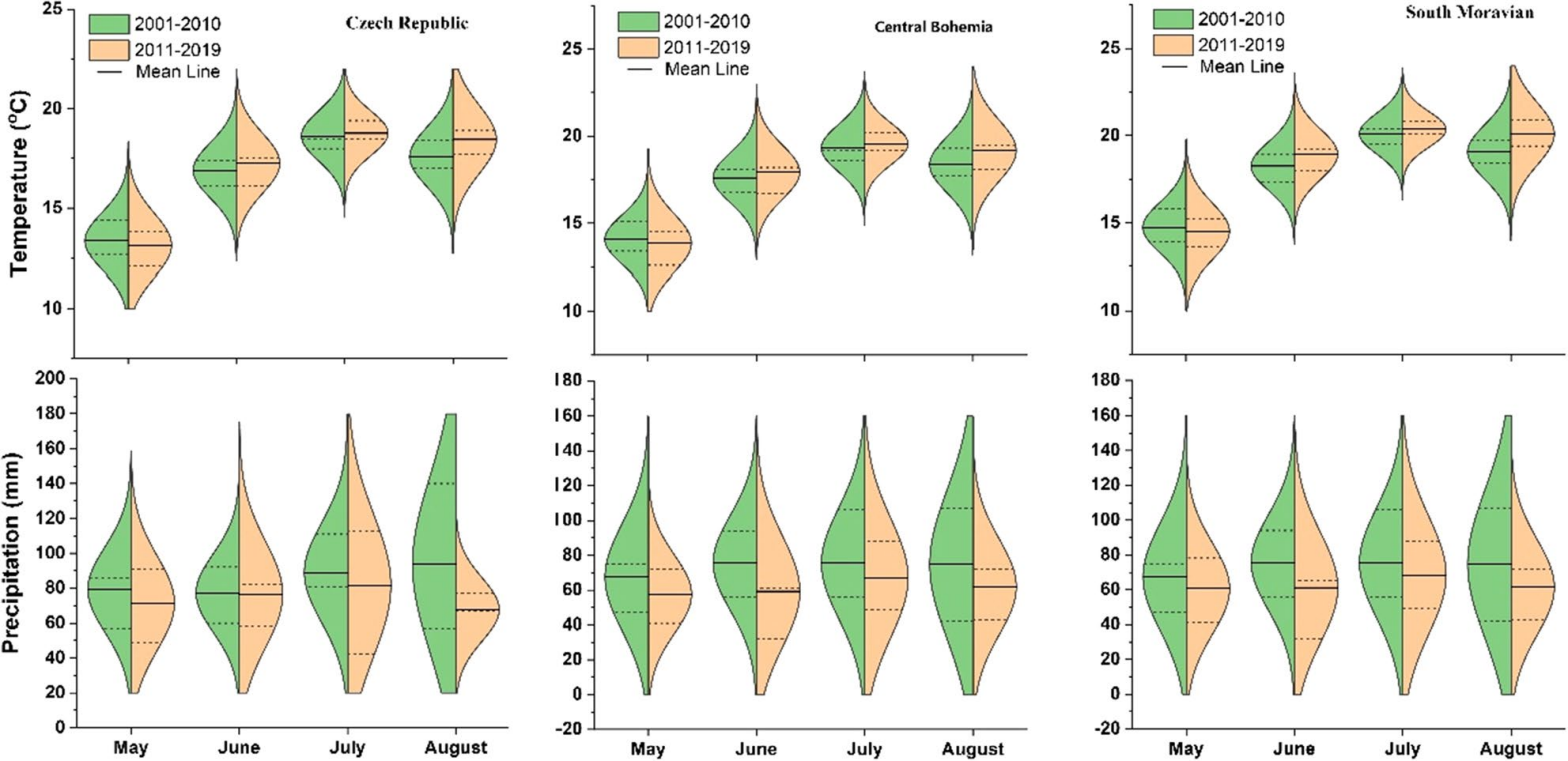
Precipitation and temperature of the maize growing season in the 2000s and 2010s in the Czech Republic, Central Bohemia, and South Moravian. The split violin plot shows both the distribution of the data and its summary statistics (mean/median and interquartile ranges).

**Figure 5:**
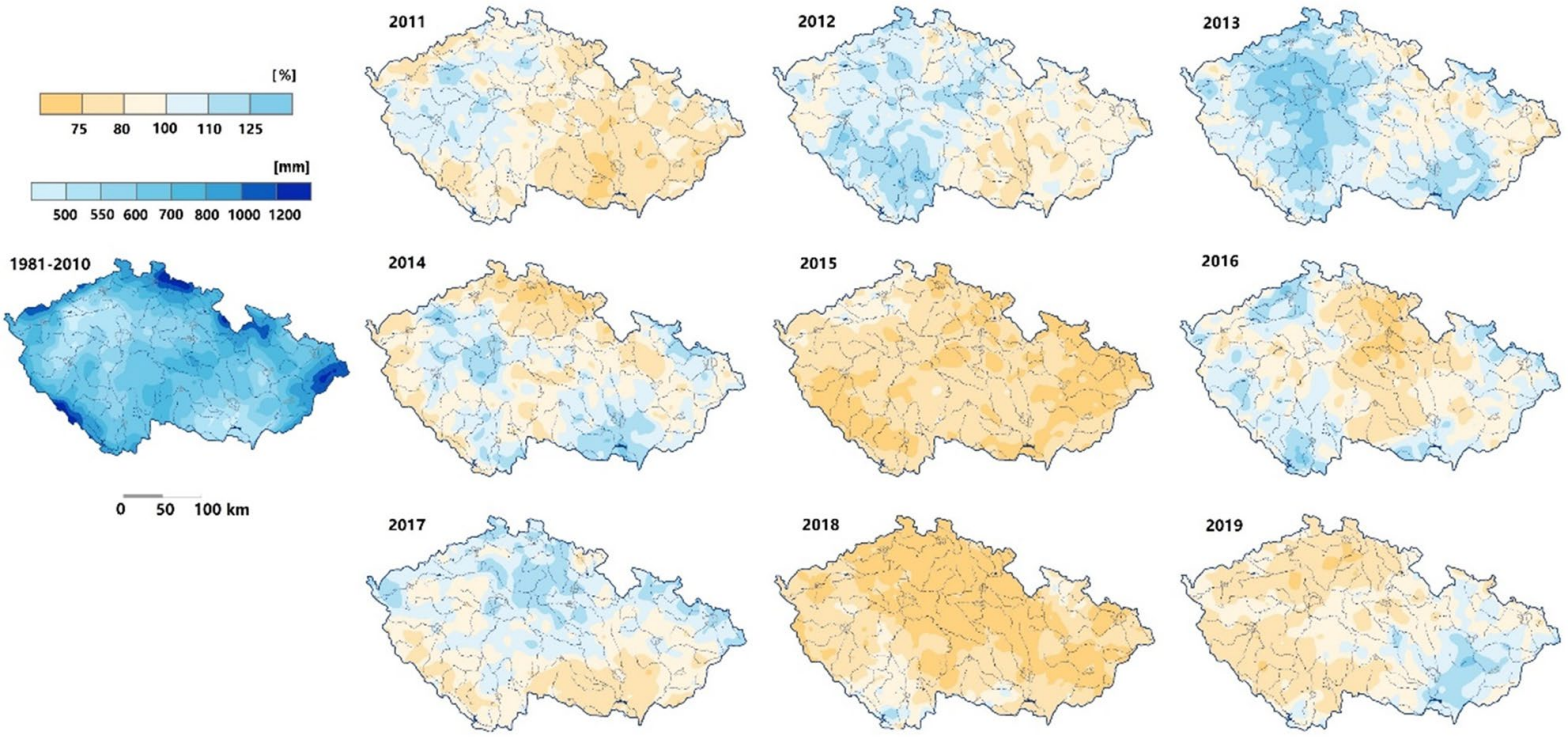
Comparison of mean annual precipitation of each year from 2011 to 2019 with the mean values of 1981–2010 ( modified from CHMI). The left map shows the average value of precipitation of 1981–2010, the individual year’s precipitation was colored based on the difference with average value of 1981–2010.

The average decade precipitation in the Czech Republic decreased from 682 mm in the 1960s to 651 mm in the 1970s, then slightly increase every decade to 722 mm in the 2000s. However, there is a sharp decrease in the 2010s to 634 mm, which is 88 mm less than the previous decade 2000s (Fig. 3). What’s more, the average precipitation during the maize growing season (May to August) was less in the 2010s than in the 2000s (Fig. 4). This phenomenon was more obvious in the Central Bohemia and South Moravian regions (Fig. 4). When we compare the annual precipitation of each year in the 2010s with the past three decades (1980–2010), most of the regions received less precipitation in the 2010s, especially in the year of 2015 and 2019 (Fig. 5).

**Figure 6:**
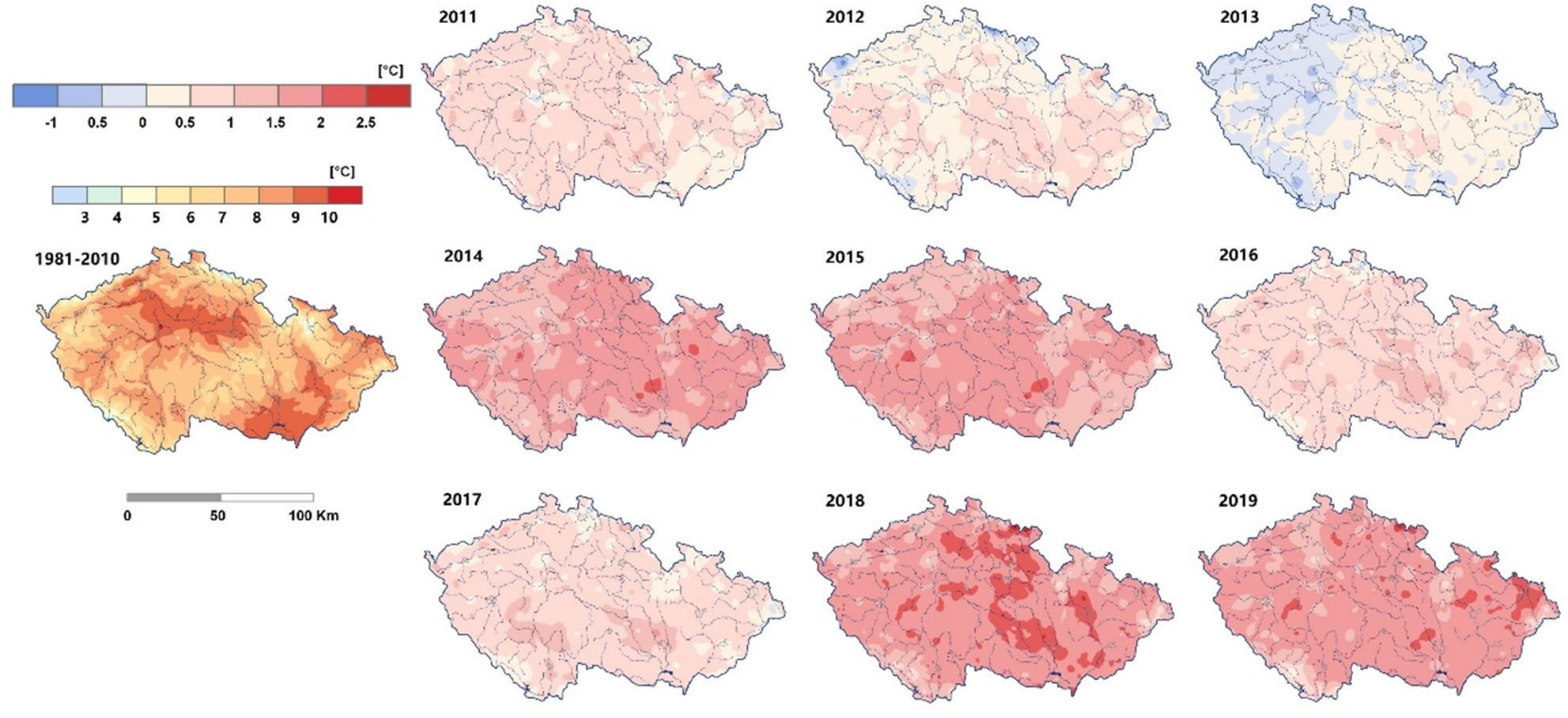
Comparison of the mean annual temperature of each year from 2011 to 2019 with the mean value of 1981–2010 ( modified from CHMI). The left map shows the average value of temperature of 1981–2010, the individual year’s precipitation was colored based on the difference with average value of 1981–2010.

The average decade temperature in the Czech Republic increased over time, from 7.1 °C in the 1960s to 8.9 °C in the 2010s (Fig. 3), which is an increased rate of 0.036 °C per year. However, there is a sharp increase of 0.7 °C in the 2010s compared to 2000s. This increase is significate (1.9 times) compare to the normal increase of 0.036 °C per year. In the maize growing season (May to August), a higher mean temperature was found (June to August) in the 2010s than in the 2000s, and a lower mean temperature was found in May in the 2010s than in the 2000s (Fig. 4). In general, the annual temperature in the 2010s is significantly higher than in the previous three decades, (Fig. 6). The extremely warm years were detected in the years 2014, 2015, 2018, and 2019.

### Effects of climate factors on maize production

Temperature is an important factor influencing the maize yield. This influence is more visible for grain maize than for silage maize (Tables 2 and 3). There is a low negative correlation (−0.34 to −0.5) between grain maize yield and the average temperature in July for 9 regions out of 14 (Table 2), and there is a low to moderate negative correlation (−0.39 to −0.51) between grain maize yield and the average temperature in August for almost all the regions (13 out 14). It’s also observed that the silage maize yield has a low negative correlation with the average temperature in July and August (6 regions for both months). In general, climate change is affected by increasing concentration of greenhouse gas, for example, CO_2_. The increase of CO_2_ has direct and indirect effects on maize production. The direct effect also called the CO_2_-fertilisation effect^19^, is because the increased CO_2_ may enhance the photosynthesis and water use efficiency, thus increase the maize growth about 14% with doubled ambient CO_2_^19,20^. The indirect effect also called the weather effect is through solar radiation, precipitation, and temperature to influence maize yield. For instance, the maize yields normally decrease with increasing temperature because of the shorter phenological phases^21^.

**Table 2:** Pearson correlation between grain maize yield rate with temperature, precipitation, and water deficit in all regions.

	Temperature				Precipitation				Water deficit			
	May	June	July	August	May	June	July	August	May	June	July	August
Prague	−0.09	−0.14	−**0.38**	−**0.44**	0.06	−0.18	**0.72**	−0.01	−0.15	0.13	−**0.64**	−0.26
Central Bohemia Region	0.18	0.09	−**0.44**	−**0.51**	−0.02	−0.25	**0.79**	0.06	0.02	0.24	−**0.71**	−**0.33**
South Bohemia Region	**0.44**	0.30	−**0.44**	−**0.39**	−0.27	−0.15	**0.69**	**0.62**	**0.32**	0.22	−**0.68**	−**0.71**
Pilsen Region	**0.40**	0.28	−**0.50**	−**0.43**	−0.11	0.04	**0.69**	**0.34**	0.20	0.03	−**0.68**	−**0.49**
Karlovy Vary Region	**0.43**	**0.33**	−**0.36**	−0.26	−0.10	−0.12	0.23	**0.38**	0.19	0.21	−0.20	−**0.44**
Ústí nad Labem Region	0.14	0.02	−**0.38**	−**0.51**	−0.05	−0.08	**0.66**	0.10	0.04	0.07	−**0.65**	−**0.31**
Liberec Region	**0.44**	0.28	−0.21	−**0.39**	−0.05	−0.15	**0.47**	0.21	0.17	0.22	−**0.38**	−**0.32**
Kralovéhradecký Region	0.18	0.04	−0.27	−**0.44**	−0.08	−0.09	**0.61**	0.13	0.09	0.15	−**0.51**	−**0.30**
Pardubice Region	**0.30**	0.13	−**0.34**	−**0.47**	−0.06	−0.01	**0.66**	0.08	0.11	0.05	−**0.59**	−**0.31**
Vysočina Region	**0.42**	0.23	−**0.38**	−**0.37**	−0.23	−0.01	**0.57**	**0.36**	0.16	0.10	−**0.58**	−**0.50**
South Moravian Region	−0.03	−0.01	−**0.35**	−**0.48**	−0.03	−0.04	**0.71**	0.02	−0.08	0.03	−**0.66**	−0.24
Zlín Region	0.17	0.03	−0.24	−**0.42**	−0.09	0.11	**0.67**	0.07	0.04	−0.06	−**0.68**	−**0.34**
Moravian-Silesian Region	**0.37**	0.19	−0.20	−**0.43**	−0.02	0.13	**0.54**	**0.42**	0.05	−0.04	−**0.58**	−**0.55**
Olomouc Region	0.11	0.05	−0.25	−**0.44**	0.02	0.02	**0.65**	0.24	0.00	−0.02	−**0.64**	−**0.45**

**Table 3:** Pearson correlation between silage maize yield rate with temperature, precipitation, and water deficit in all regions.

	Temperature				Precipitation				Water deficit			
	May	June	July	August	May	June	July	August	May	June	July	August
Prague	−0.16	−0.27	−**0.32**	−0.20	0.04	0.18	**0.43**	−0.25	−0.08	−0.17	−**0.47**	0.05
Central Bohemia Region	−0.11	−0.29	−**0.32**	−0.26	0.00	0.10	**0.50**	−0.19	−0.04	−0.10	−**0.50**	−0.04
South Bohemia Region	−0.01	−0.21	−**0.31**	−**0.35**	−0.01	−0.13	**0.70**	0.16	−0.05	0.10	−**0.69**	−0.33
Pilsen Region	0.03	−0.26	−**0.31**	−**0.31**	−0.18	0.11	**0.57**	0.05	0.22	−0.07	−**0.56**	−0.23
Karlovy Vary Region	0.16	−0.25	−**0.30**	−**0.32**	−**0.32**	0.04	**0.36**	−0.04	**0.32**	0.07	−**0.37**	−0.15
Ústí nad Labem Region	−0.05	−0.30	−**0.32**	−**0.31**	0.04	**0.39**	**0.36**	−0.10	−0.08	−**0.39**	−**0.49**	−0.03
Liberec Region	0.04	−0.29	−0.24	−**0.39**	−0.01	**0.31**	**0.56**	−0.09	0.03	−0.26	−**0.59**	−0.05
Kralovéhradecký Region	−0.03	−0.26	−0.26	−0.24	−0.07	**0.30**	**0.47**	−**0.35**	0.04	−**0.33**	−**0.45**	0.29
Pardubice Region	−0.05	−0.26	−0.25	−0.26	−0.14	0.20	**0.46**	−0.27	0.10	−0.13	−**0.43**	0.08
Vysočina Region	0.02	−0.17	−0.21	−**0.31**	−0.29	0.06	**0.56**	−0.05	0.22	0.04	−**0.50**	−0.19
South Moravian Region	−0.15	−0.14	−0.26	−0.19	−0.15	−0.06	**0.44**	−0.22	0.22	0.04	−**0.50**	−0.19
Zlín Region	−0.08	−0.15	−0.21	−0.22	−0.22	0.07	**0.38**	−0.16	0.07	−0.04	−**0.42**	−0.01
Moravian-Silesian Region	−0.04	−0.16	−0.20	−0.29	−0.12	0.20	**0.35**	0.02	0.00	−0.04	−**0.44**	−0.23
Olomouc Region	−0.08	−0.17	−0.26	−0.21	−0.18	0.17	**0.41**	−0.20	0.16	−0.08	−**0.41**	0.01

Precipitation is another important factor influencing the maize yield. Both grain and silage maize yield rate showed a positive correlation with precipitation in July (Tables 2 and 3), and the correlation is stronger for grain than for silage. The grain maize yield rate had a moderate to high positive correlation (0.54–0.79) with precipitation in July for almost all the regions (expect Karlovy Vary Region). The silage maize yield rate had a low to moderate positive correlation (0.35–0.70) with precipitation in July for all the regions. The precipitation from other months (May, June, and August) only have a low or negligible correlation with maize yield rate for both grain and silage (Tables 2 and 3). Our results are being consistent with the previous results that relatively high correlations between precipitation in July with silage yield, which explained 64% of the observed variability in the average silage yields of maize^8^. In principle, the effect of precipitation may be either positive or negative. Positive correlation may happen if precipitation reduces the existing water stress, and negative correlation may because of the intensified nitrogen leaching by the excessive precipitation^21^. However, the relationships between maize yields and climate change characteristics can be different due to individual sites depending on the present climate conditions. For example, the relationship between precipitation (total in the growing season) and maize yield was stronger in the southeastern than in the northeastern U.S., but the relationship between critical month precipitation and maize yield was stronger in the northeastern than in the southeastern U.S.^22^. Nevertheless, the Czech Republic is supposed to face a decrease in spring and summer precipitation in most of the regions^23^.

Water deficit exhibited a negative correlation with both maize yield rate (Tables 2 and 3), and its influence mainly in July for silage but both in July and August for grain. There is a low to high negative correlation (−0.38 to −0.71) between grain maize yield rate and average water deficit in July for almost all regions (expect Karlovy Vary Region), and also a low to high negative correlation (−0.30 to −0.71) between grain maize yield rate and average water deficit in August for 12 regions out of 14. There is a low to moderate negative correlation (−0.37 to −0.69) between silage maize yield rate and average water deficit in July for all the regions. Therefore, the critical month for water deficit of both grain and silage is July. What’s more, the relationship between maize yield and precipitation showed that moisture shortage rather than excess determined maize yield in the Eastern United States^22^.

Water deficit is one of the most significant stress factors in crop production globally^24^, and it leads to significant yield reduction. The Czech Republic is not generally considered as a drought-prone region in Europe, but there are more and more droughts were recorded, for example, 2000, 2001, 2003, 2014, 2015, and 2018. It was reported that the highest water deficit was recorded for maize growing areas from the field block scale in the Czech Republic due to low precipitation and high water requirements, and about 39.6% (9745.5 km^2^) of silage maize growing area was effected^25^. The droughts had a profound effect on national and regional agricultural production in the Czech Republic, with yields being consistently lower than in normal years^26^. There is a statistically significant correlation (p < 0.05) between the sum of Palmer’s Z-index for maize, and the yield departure is 48%^27^. Unfortunately, the tendency to more intensive dry episodes may happen more frequently in the Czech Republic because it’s driven by temperature increase and precipitation decrease, and it already very prominent since 2010 (Fig. 3).

However, except the effect of climate change on maize production, some other technological boundaries also demonstrate their influence on maize production^28^. Lower estimations of maize productivity and technological dependability of current maize cultivars can identify with production techniques predominately pointed toward expanding yield. It is all around recorded in France and the United Kingdom, where a reformist expansion in maize yields by 0.12 t/ha each year was identified with a decline in maize production during the most recent 50 years. In the Czech Republic, maize yield is mostly influenced by location, nitrogen fertilization and year with 120 kg N/ha being considered a sufficient application^29^. However, yield was also related to the percentage of perennial legumes used, particularly when low rates of nutrients were applied (lower than 100 kg NPK ha^−1^ of arable land). The influence of varieties was relatively low in comparison to the influences of location, year, nitrogen application, use of growth regulators and fungicides.

Furthermore, some multi-temporal-scale index that quantifies persistent anomalies in the local soil moisture balance may better identify the effects of climate factors on maize production. For example, the combined stress index (CSI) which integrates the standardised precipitation evapotranspiration index (SPEI) and the heat magnitude day index (HMD) was used to predict maize production^30^. In which, the SPEI is a multi-temporal-scale index and it is able to capture the impact of drought on agricultural production^31^. This should be taken into account for future research, for example, combined with water deficit.

### Profit and its relationship with temperature, precipitation, and water deficit

Farmer’s profit from maize was calculated only for grain because silage was most used by the farmers. The heatmap shows that profit has a moderate to high positive correlation to grain yield rate (Table 4). The average temperature in July and August have a low to moderate negative influence on profit. Precipitation of the whole growing season (May to August) has a low to moderate positive influence on profit, with the negative effect from May and June but the positive effect from July and August. In general, water deficit hurts the farm’s profit, and the water deficit in July and August have a moderate to high negative influence on profit, while the effects from May and June are neglective. Water deficit can be express as drought and it is an issue for all European Union (EU) countries. It was reported that 15% of the EU territory and 17% of the EU population from 2006 to 2010 have been affected with economic losses of over € 100 billion^32^.

**Table 4:** Pearson correlation between profit with temperature, precipitation, and water deficit.

	Temperature					Precipitation					Water deficit				
	May–Aug	May	June	July	August	May–Aug	May	June	July	August	May–Aug	May	June	July	August
Prague	−0.41	−0.12	0.09	−0.44	−0.48	0.46	0.20	0.03	0.52	0.21	−0.47	−0.23	−0.04	−0.52	−0.36
Central Bohemia	−0.25	0.11	0.27	−0.41	−0.47	0.41	0.10	−0.14	0.58	0.26	−0.40	−0.08	0.16	−0.53	−0.42
South Bohemia	0.30	0.44	0.39	0.00	−0.02	0.51	−0.35	−0.17	0.23	0.82	−0.44	0.38	0.29	−0.21	−0.80
Pilsen	0.16	0.38	0.39	−0.20	−0.08	0.36	−0.12	−0.15	0.37	0.47	−0.25	0.21	0.24	−0.32	−0.48
Karlovy Vary	0.30	0.23	0.22	0.00	0.25	0.14	−0.19	−0.05	−0.22	0.57	−0.04	0.21	0.14	0.17	−0.35
Ústí nad Labem	−0.40	0.03	0.05	−0.45	−0.53	0.32	0.00	−0.24	0.76	0.09	−0.31	−0.02	0.17	−0.71	−0.33
Liberec	0.10	0.05	−0.02	0.00	0.18	0.11	−0.10	−0.23	0.16	0.22	−0.02	0.10	0.28	−0.13	−0.19
Kralovéhradecký	−0.17	0.13	0.24	−0.29	−0.40	0.58	0.12	−0.03	0.46	0.42	−0.56	−0.06	−0.02	−0.42	−0.55
Pardubice	−0.01	0.30	0.32	−0.21	−0.32	0.45	0.06	0.00	0.44	0.30	−0.42	0.03	0.06	−0.41	−0.42
Vysočina	0.31	0.32	0.25	0.12	0.15	0.27	−0.23	−0.07	0.02	0.58	−0.16	0.25	0.17	−0.01	−0.48
South Moravian	−0.32	−0.03	0.05	−0.42	−0.44	0.30	0.16	0.34	0.20	0.00	−0.32	−0.17	−0.29	−0.29	−0.10
Zlín	−0.09	0.23	0.11	−0.22	−0.33	0.43	0.07	0.46	0.29	0.14	−0.45	−0.07	−0.37	−0.46	−0.30
Moravian-Silesian	0.23	0.41	0.28	0.03	−0.12	0.27	0.08	0.07	0.18	0.39	−0.32	−0.04	0.01	−0.22	−0.37
Olomouc	−0.17	0.13	0.17	−0.26	−0.41	0.49	0.24	0.12	0.30	0.43	−0.49	−0.15	−0.21	−0.40	−0.47
CZ	0.15	0.25	0.31	−0.01	−0.06	0.20	−0.01	−0.02	0.19	0.23	−0.12	0.06	0.10	−0.16	−0.22

**Figure 7:**
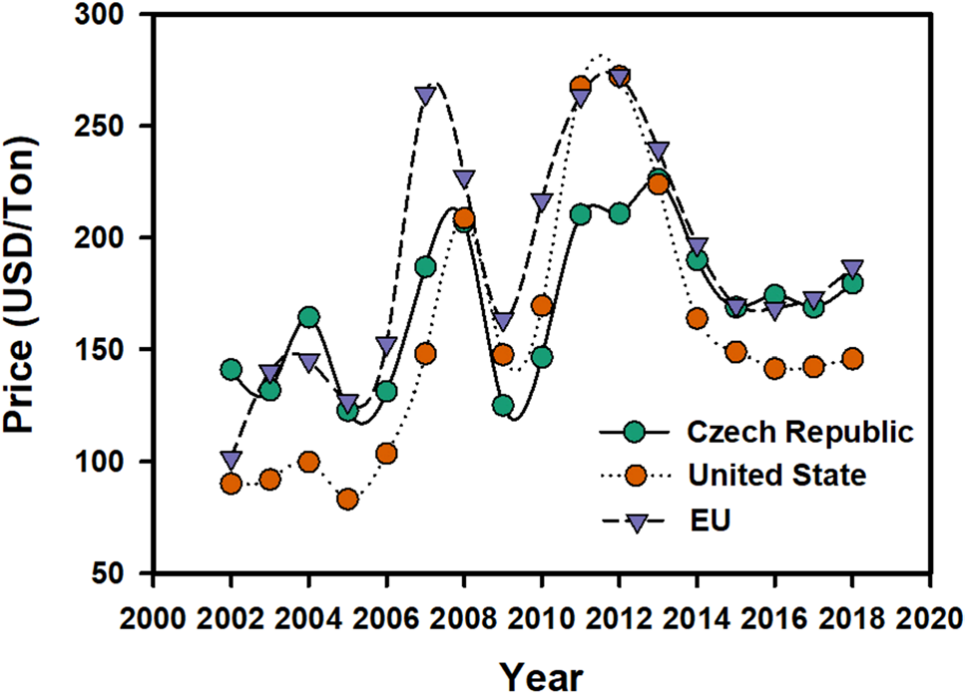
Maize prices in the Czech Republic, EU and US CME market (based on CZSO ( *source*: https://vdb.czso.cz/vdbvo2/faces/en/index.jsf?page=vystup-objekt&z=T&f=TABULKA&katalog=31785&pvo=CEN02AA&evo=v874_!_CEN02AA-R_1cme.com), FAOSTAT (source: ) and CME data (#/)).

From the economic aspect, the regional maize prices are highly correlated with the world markets (Fig. 7). There is a high correlation in the long-run (0.8 for Czech to EU prices, and 0.823 for Czech to US CME prices, and 0.883 for EU to USD CME prices). At the beginning of the observed period, the maize prices followed the increasing trend of other commodities which was started by increasing crude oil prices in 2002^33^. Since 2006 maize price started to peak to its historical maximums. The demand was particularly led by the increasing production of bio-ethanol in the United States^34^. The relationship^35^ and asymmetric volatility transmission between maize and ethanol prices has been also found as one of the key variables^36^. The decrease in 2009 was common for most of the commodity markets and it is related to the financial crisis in 2008^37^. The maize markets peaked to its all-time highs in 2012 and 2013. The maize prices followed again the other commodities including the crude oil and the decision of Saudi Arabia to remove the extraction limits on crude oil production which brought the overproduction and started the price decrease^38^. There are many factors which affect maize markets. The widely discussed bio-ethanol production and policies supporting the bio-fuel usage can contribute to the higher effectivity in corn production and yield, as there comes more investments in technologies from farmers^39^.

From the environmental and climate aspects, agriculture is by a long shot the greatest worldwide user of freshwater resources and subsequently exceptionally helpless against environmental and climate change^40^. Climatic conditions straightforwardly influence global agriculture trade and the water resources expected to keep a steady production in numerous territories of Europe^41^. The pressure forced by environmental and climate change on agriculture and water will upgrade existing provincial incongruities in the Czech Republic and other European countries^42,43^.

## Conclusion

Even there are other factors (e.g. fertility level, plant management, and insect, disease, and weed pressures, etc.) that can affect the production of maize, there is no doubt that precipitation is one of the most important factors controlling maize yield both for grain and silage. Furthermore, water deficit with the combination of precipitation and temperature (which is one of the most important parameters for climate change) is even more significant and critical for maize (both for grain and silage) production. Meanwhile, water deficit rather than water surplus determined maize yield in the Czech Republic. As a result, farmer’s profit from maize was significantly associated with water deficit, precipitation, and temperature, and it’s decreasing under the changing climate (warmer and dryer) in the Czech Republic. Therefore, more technology and management approaches (e.g. appropriate tillage, and agricultural water management) are needed to cope with climate change.

## References

[1] FAO. Cereal markets to remain well supplied in 2020/21. http://www.fao.org/worldfoodsituation/csdb/en/ (2020).

[2] OECD/FAO. *OECD-FAO Agricultural Outlook 2020–2029*. (OECD Publishing, 2020).

[3] Olesen JE (2007). Uncertainties in projected impacts of climate change on European agriculture and terrestrial ecosystems based on scenarios from regional climate models. Clim. Change.

[4] Reidsma P, Ewert F, Lansink AO, Leemans R (2010). Adaptation to climate change and climate variability in European agriculture: The importance of farm level responses. Eur. J. Agron..

[5] Darwin R, Kennedy D (2000). Economic effects of CO2 fertilization of crops: transforming changes in yield into changes in supply. Environ. Model. Assess..

[6] Chloupek O, Hrstkova P, Schweigert P (2004). Yield and its stability, crop diversity, adaptability and response to climate change, weather and fertilisation over 75 years in the Czech Republic in comparison to some European countries. Field Crop. Res..

[7] Trademap. List of products imported by Czech Republic, detailed products in the following category: 100590 Maize (excluding seed for sowing). https://www.trademap.org/ (2020).

[8] Žalud Z (2017). Impacts of water availability and drought on maize yield – A comparison of 16 indicators. Agric. Water Manag..

[9] Trnka M (2012). Could the changes in regional crop yields be a pointer of climatic change?. Agric. For. Meteorol..

[10] Trnka M (2016). Assessing the combined hazards of drought, soil erosion and local flooding on agricultural land: A Czech case study. Clim. Res..

[11] Semenov MA, Shewry PR (2011). Modelling predicts that heat stress, not drought, will increase vulnerability of wheat in Europe. Sci. Rep..

[12] Kolář P, Trnka M, Brázdil R, Hlavinka P (2014). Influence of climatic factors on the low yields of spring barley and winter wheat in Southern Moravia (Czech Republic) during the 1961–2007 period. Theoret. Appl. Climatol..

[13] Partridge TF (2019). Mid-20th century warming hole boosts US maize yields. Environ. Res. Lett..

[14] IndexMundi. European Union (EU-27) Corn Yield by Year. https://www.indexmundi.com/agriculture/?country=eu&commodity=corn&graph=yield (2020).

[15] Tigchelaar M, Battisti DS, Naylor RL, Ray DK (2018). Future warming increases probability of globally synchronized maize production shocks. Proc. Natl. Acad. Sci. USA.

[16] Zampieri M (2019). When will current climate extremes affecting maize production become the norm?. Earth’s Fut..

[17] Chloupek O, Hrstkova P, Schweigert P (2004). Yield and its stability, crop diversity, adaptability and response to climate change, weather and fertilisation over 75 years in the Czech Republic in comparison to some European countries. Field Crop Res.

[18] Pulkrábek, J. *et al.* Regional food and feed self-sufficiency related to climate change and animal density—a case study from the Czech Republic. *Plant Soil Environ.* (2019). 10.17221/190/2019-PSE.

[19] Dhakhwa GB, Campbell CL, LeDuc SK, Cooler EJ (1997). Maize growth: Assessing the effects of global warming and CO2 fertilization with crop models. Agric. For. Meteorol..

[20] Poorter H (1993). Interspecific variation in the growth response of plants to an elevated ambient CO2 concentration. Vegetatio.

[21] Brown RA, Rosenberg NJ (1997). Sensitivity of crop yield and water use to change in a range of climatic factors and CO2 concentrations: A simulation study applying EPIC to the central United States. Agric. For. Meteorol..

[22] Huang C, Duiker SW, Deng L, Fang C, Zeng W (2015). Influence of precipitation on maize yield in the eastern United States. Sustainability (Switzerland).

[23] Hanel M, Vizina A, MácA P, Pavlásek J (2012). A multi-model assessment of climate change impact on hydrological regime in the Czech Republic. J. Hydrol. Hydromech..

[24] Lobell DB, Field CB (2007). Global scale climate-crop yield relationships and the impacts of recent warming. Environ. Res. Lett..

[25] Duffková R, Holub J, Fucík P, Rožnovskỳ J, Novotnỳ I (2019). Long-term water balance of selected field crops in different agricultural regions of the czech republic using fao-56 and soil hydrological approaches. Sustainability (Switzerland).

[26] Brázdil R (2009). Variability of droughts in the Czech Republic, 1881–2006. Theoret. Appl. Climatol..

[27] Hlavinka P (2009). Effect of drought on yield variability of key crops in Czech Republic. Agric. For. Meteorol..

[28] Triboi E, Martre P, Girousse C, Ravel C, Triboi-Blondel AM (2006). Unravelling environmental and genetic relationships between grain yield and nitrogen concentration for wheat. Eur. J. Agron..

[29] Vrkoc, F. Contribution of some factors to the development of crop production in the CSFR. *Scientia Agriculturae Bohemoslovaca UVTIZ* (1992).

[30] Ceglar A (2018). Land-surface initialisation improves seasonal climate prediction skill for maize yield forecast OPEN. Sci. Rep..

[31] Library WO (2015). Candidate Distributions for Climatological Drought Indices (SPI and SPEI). Int. J. Climatol..

[32] Spinoni, J., Naumann, G., Vogt, J. & Barbosa, P. *Meteorological Droughts in Europe: Events and Impacts – Past Trends and Future Projections*. (2016). 10.2788/450449.

[33] Good, D. *USDA Reports to Support Corn Prices*. https://farmdocdaily.illinois.edu/2015/01/usda-reports-to-support-corn-prices.html (2015).

[34] Babcock BA, Fabiosa JF (2011). The impact of ethanol and ethanol subsidies on corn prices: revisiting history. CARD Policy Briefs..

[35] Dutta A, Bouri E, Junttila J, Uddin GS (2018). Does corn market uncertainty impact the US ethanol prices?. GCB Bioenergy.

[36] Saghaian S, Nemati M, Walters C, Chen B (2018). Asymmetric price volatility transmission between US Biofuel, Corn, and Oil Markets. J. Agric. Resource Econ..

[37] Zhang D, Broadstock DC (2020). Global financial crisis and rising connectedness in the international commodity markets. Int.Rev. Financ. Anal..

[38] Krane J (2015). A refined approach: Saudi Arabia moves beyond crude. Energy Policy.

[39] Szabó Z (2019). Can biofuel policies reduce uncertainty and increase agricultural yields through stimulating investments?. Biofuels, Bioprod. Biorefin..

[40] Iglesias A (2006). Challenges to Manage the Risk of Water Scarcity and Climate Change in the Mediterranean..

[41] Iglesias, A., Rosenzweig, C. & Pereira, D. *Agricultural impacts of climate change in Spain: developing tools for a spatial analysis*. *es (A. Iglesias). Global Environmental Change* vol. 10 (2000).

